# P-2115. Clinical Presentation and Outcome of *Tuberculosis* in Chronic Kidney Disease Stage 4 and 5 from a high TB burden Country

**DOI:** 10.1093/ofid/ofae631.2271

**Published:** 2025-01-29

**Authors:** Rohama Samar, Sunil Dodani, Asma Nasim, Zaheer Udin

**Affiliations:** Sindh Institute of Urology and Transplantation, Karachi, Sindh, Pakistan; Sindh Institute of Urology and Transplantation, Karachi, Sindh, Pakistan; Sindh Institute of Urology and Transplantation, Karachi, Sindh, Pakistan; Sindh Institute of Urology and Transplantation, Karachi, Sindh, Pakistan

## Abstract

**Background:**

Diagnosis and management of Tuberculosis (TB) in chronic kidney disease (CKD) is challenging. Our aim is to study clinical presentation and outcome in patients with stage 4 and 5 CKD from a high TB burden country.

**Figure:**
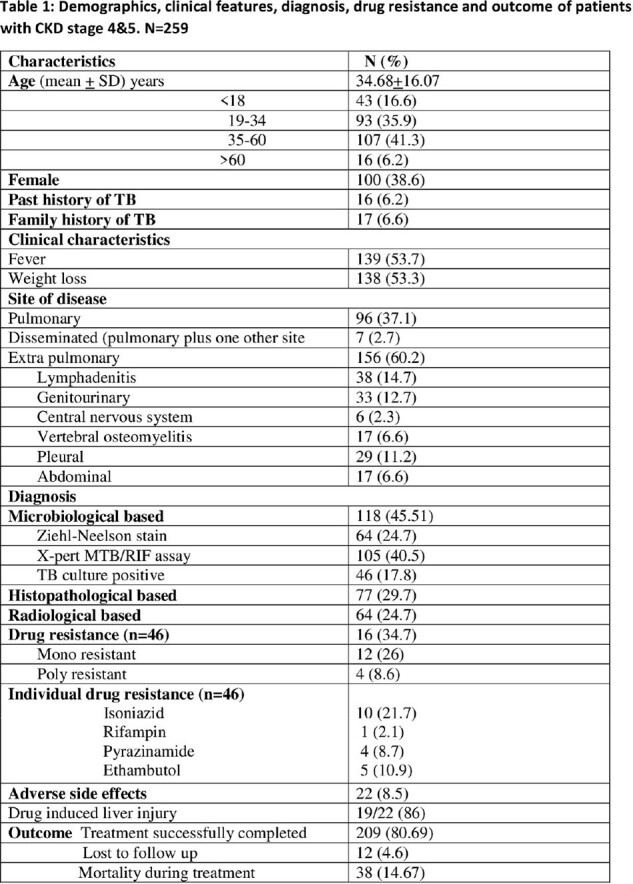

**Methods:**

All patients registered from May 2016 to June 2020 were included. Rifampicin resistance on GeneXpert test was excluded. Patients with CKD stage 4 and 5 were studied for demographics, TB history, clinical features, diagnoses, treatment success and mortality. CKD stage 4 and 5 were compared with other patients registered at the TB treatment center.

**Figure:**
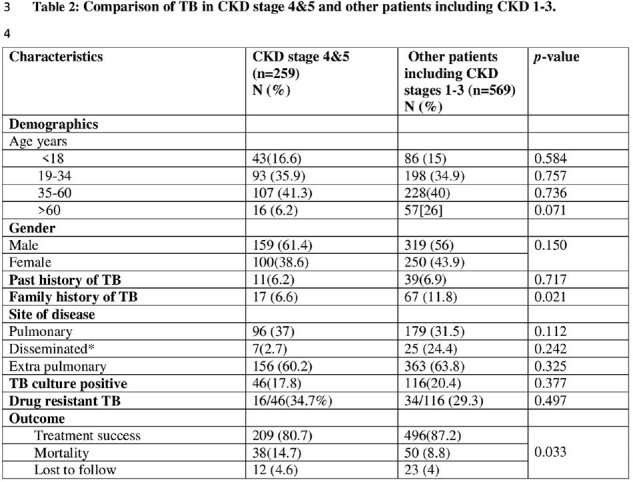

**Results:**

Out of 828, 259 (31%) had CKD stage 4 and 5. Out of 259, 156 (60%) had extra-pulmonary TB (EPTB). Microbiological diagnosis done in 118 (45.51%), 25% in EPTB and 72.9% in pulmonary TB (PTB). TB culture was positive in 46 (17.8%), Isoniazid resistance [10] 21.7%. Treatment success was 80.7%. PTB was significantly associated with mortality (p=0.031). In CKD stage 4 and 5 treatment success was significantly lower with high mortality (p=0.033).

**Figure:**
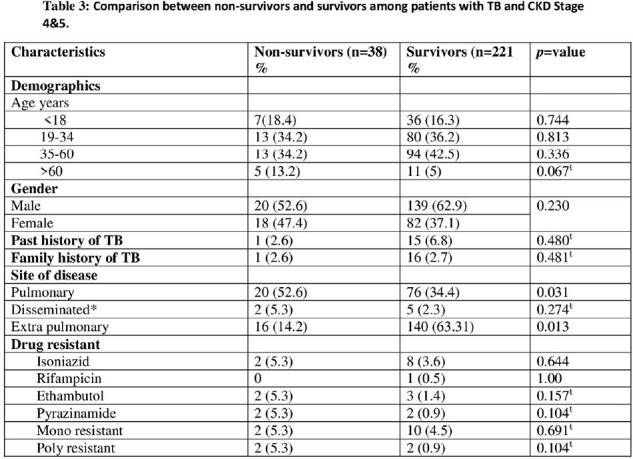

**Conclusion:**

In CKD stage 4 and 5, EPTB is the most common presentation. Microbiological diagnosis could be achieved in one -fourth of EPTB. There is high INH resistance. The treatment success is low with high mortality and PTB is a significant risk factor for mortality.

**Disclosures:**

All Authors: No reported disclosures

